# A Case of Gingival Candidiasis with Bone Destruction on Gastric Cancer Patient Receiving Cytotoxic Chemotherapy

**DOI:** 10.1155/2014/145394

**Published:** 2014-12-29

**Authors:** Seungtaek Lim, Tae-jun Kil, Hye Ryun Kim, Seonhui Han, Sun Young Rha

**Affiliations:** ^1^Department of Internal Medicine, Konyang University Hospital, Gasuwon-dong, Seo-gu, Daejeon 302-718, Republic of Korea; ^2^Department of Oral and Maxillofacial Surgery, Yonsei University College of Dentistry, 50-1 Yonsei-ro, Seodaemun-gu, Seoul 120-752, Republic of Korea; ^3^Division of Medical Oncology, Department of Internal Medicine, Yonsei University College of Medicine, 50 Yonsei-ro, Seodaemun-gu, Seoul 120-752, Republic of Korea; ^4^Yonsei Cancer Center, Yonsei Cancer Research Institute, 50 Yonsei-ro, Seodaemun-gu, Seoul 120-752, Republic of Korea; ^5^Brain Korea 21 Project for Medical Science, Yonsei University College of Medicine, 50 Yonsei-ro, Seodaemun-gu, Seoul 120-752, Republic of Korea; ^6^Department of Oral Pathology, Yonsei University College of Dentistry, 50-1 Yonsei-ro, Seodaemun-gu, Seoul 120-752, Republic of Korea

## Abstract

We herein report a case of gingival candidiasis in an advanced gastric cancer patient while receiving palliative cytotoxic chemotherapy. A 46-year-old male patient admitted to our hospital for known advanced gastric cancer with newly developed multiple liver metastases. While receiving 2nd line cytotoxic chemotherapy with 5FU, leucovorin, and paclitxel, he complained of gingival swelling accompanied by pain and whitish plaque. Due to lack of response to the conservative oral care, incisional biopsy of gingiva was done and the pathology confirmed gingival candidiasis. Although the lesion healed apparently after two-week antifungal therapy, pain as well as bony destruction remains. By presenting this case report, we intend to emphasize the immunocompromising effect of cancer while being on systemic chemotherapy.

## 1. Introduction

Gastric cancer is the second in all causes of cancer related deaths worldwide with higher prevalence in East Asia [1]. The prognosis is extremely poor in patients with unresectable or metastatic gastric cancer and palliative chemotherapy is the treatment of choice for them [2]. Since the aim of chemotherapy in this setting is to prolong the survival period [3] and to palliate symptoms, quality of life is one of the important issues. Therefore, chemotherapy induced toxicity should be well balanced with its efficacy [4]. Despite the development of new generation of chemotherapeutic agents including molecular targeted drugs with less toxicity, still the conventional cytotoxic chemotherapeutic agents have the major role in palliative treatment of gastric cancer. So, the complications from the cytotoxic chemotherapy are still of major concern. Oral complications are one of the commonest symptomatic and troublesome problems resulting in difficulties in eating, poor oral intake with weight loss, and poor performance [5]. The subsequent nutritional impairment, in turn, has a negative impact on the host immune system, making patients vulnerable to the variable infectious organisms. Candida is one of the normal floras residing in the oral cavity, but oral candidiasis rarely develops in the gingiva among those with intact immune system. In addition, gingival candidiasis has been reported almost exclusively in HIV-seropositive patients and known as a surrogate marker for the immunocompromised status. Here we report a case of gastric cancer patient who suffered from gingival candidiasis which has progressed to periodontitis resulting in permanent bony destruction. By this case report, we intend to emphasize the immune compromising effect of cancer, especially when they are treated with the cytotoxic chemotherapy agents.

## 2. Case Report

A 46-year-old male patient was referred to our hospital with complaint of epigastric discomfort for 6 months. As endoscopy and abdominopelvic computed tomography (CT) showed advanced gastric cancer at lesser curvature, lower body of stomach without distant metastasis, he underwent radical subtotal gastrectomy. The final pathological diagnosis was poorly differentiated tubular adenocarcinoma without vascular or lymphatic invasion with T2N2M0, stage IIB according to TNM 7th edition. However, one month later when he admitted for adjuvant chemotherapy, the CT scan showed multiple liver metastases without evidence of other site metastases or carcinomatosis. He was enrolled to randomized, placebo controlled phase III clinical trial exploring the efficacy of bevacizumab in combination with capecitabine and cisplatin. After eleven cycles of chemotherapies, follow-up CT scan revealed further progression of liver metastasis. Since the patient's performance status was Eastern Cooperative Oncology Group (ECOG) 1, we decided to treat him with 2nd line palliative chemotherapy of FL-Taxol (infusional fluorouracil with leucovorin and paclitaxel).

**Figure 1:**
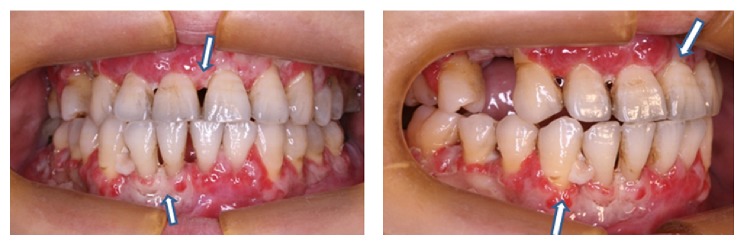
Generalized gingival swelling and redness with desquamation of necrotic tissue is observed (arrow).

After the 2nd cycles of FL-Taxol chemotherapy, he visited outpatient clinic for excessive oral pain resulting in poor oral intake lasting for one week. On inspection, the gingiva swelled and whitish plaque was covered on it. There was a foul odor from the lesion, too. At that time, there was no evidence of other chemotherapy related toxicities such as bone marrow suppression, diarrhea, or neuropathies. Chest X-ray was normal, and he was in afebrile status. With the impression of chemotherapy-induced localized gingivitis, routine oral care such as scaling, tooth brushing instruction was done. However, since desquamation continued and symptom did not subside despite conventional oral care for 2 weeks, he was referred to the oral and maxillofacial surgeon. Incisional biopsy was done to differentiate gingival infection or metastatic lesion (Figure 1).

**Figure 2:**
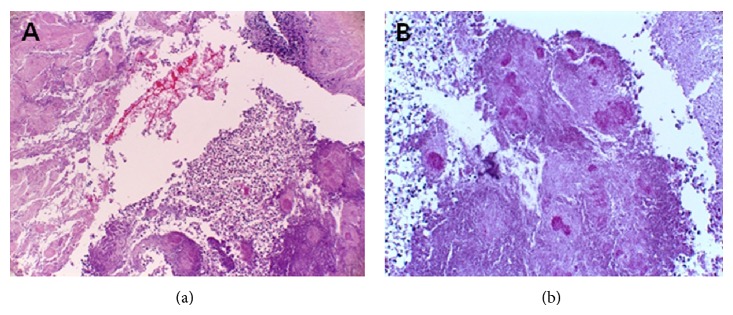
(a) Necrotic tissue with chronic nonspecific inflammation (H-E stain, ×100). (b) PAS stain reveals candida hyphae (periodic acid-Schiff stain, ×200).

**Figure 3:**
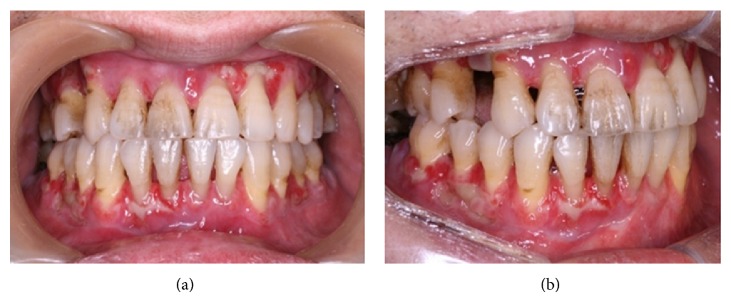
An apparent improvement, with a decreased swelling and necrotic tissue, is observed after a two-week antifungal therapy.

The pathologic finding revealed necrotic tissue with candidiasis (Figure 2). The serologic test for human immunodeficiency virus was negative, and there was no evidence of systemic involvement of candidiasis other than gingiva. We started oral fluconazole 100 mg daily with nystatin suspension. When he visited outpatient clinic two weeks later, the apparent gingival lesion nearly disappeared (Figure 3). But, excessive oral pain and bony destruction still persisted. During the follow-up period, the necrotic alveolar bone was exposed due to desquamation of the necrotized gingiva. The necrotic gingiva and sequestra were removed as needed.

**Figure 4:**
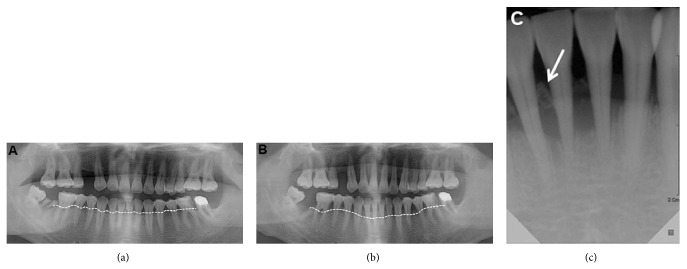
(a) Initial panoramic view (September 2, 2009). The dotted line denotes the marginal bone level. (b), (c) Panoramic and periapical view 2 months after treatment (November 19, 2009). Note the marginal bone loss and the sequestrum at the lower anterior teeth area (arrow). Also note that the radices of the second right lower molar have been removed.

Two months later, the gingival lesion was nearly healed, but the radiographic examinations revealed severe marginal bone loss at upper and lower incisor due to destructive change by the previous inflammation (Figure 4). Loss of the attached gingiva and marginal bone resulted in an increased mobility of the overall teeth, especially the lower anterior teeth. Hence, additional visits to the dentist for dental extractions and prosthodontic rehabilitation were required, but poor performance status hindered him from getting further dental care.

**Figure 5:**
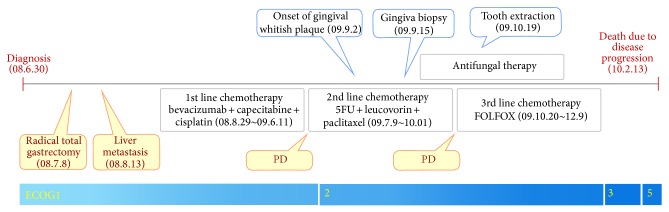
Summary of patient progress.

While the patient received dental care, with improved general condition, he resumed 3rd cycle of chemotherapy. Since CT scan after 4 cycles FL/Taxol showed further progression of liver metastasis, regimen change to FOLFOX was done. After 4 cycles of FOLFOX chemotherapy, CT scan revealed disease progression, and the patient stopped further chemotherapy because of poor performance. He expired due to disease progression on 13th February, 2010 (Figure 5).

## 3. Discussion 

For metastatic advanced gastric cancer, palliative chemotherapy is the mainstay of treatment. Since the combination of TS-1, an oral derivative of 5-fluorouracil (5-FU), with cisplatin demonstrated overall survival over 12 months in SPIRIT trial, 5-FU analogues with platinum agent has been generally accepted as standard first line regimen for the patients with recurrent or metastatic gastric cancer [6].

Various organisms are responsible for oral infection. In an earlier literature investigating the incidence of oral infection, the frequency rates were 9.7% for the total population [7]. Almost all (93.8%) of the infections were caused by single organism, and only 6.2% were polymicrobial, of which 68.9% isolates were fungal. Gram negative bacilli and Gram positive cocci were isolated from 10.7% and 9.7%, respectively. While several species of candida reside in the mouth as normal flora, opportunistic infection by them can occur when host immune system is compromised [8]. Among them,* Candida albicans* is causative organism for over 90% of the fungal infections encountered in cancer patients [7].

Although candida species are frequently isolated in the subgingival tissue, gingival candidiasis rarely developed in the individual with intact immune system [9, 10]. It occurs almost exclusively in HIV-seropositive or other immunocompromised patients and presents as erythema of the attached gingiva, referred to as HIV-associated gingivitis or linear gingival erythema [10]. Periodontitis is caused by the extension of infection from the gingiva to the periodontium and resorption of alveolar bone, resulting in loosening and loss of teeth and deep dental pain. In HIV-seropositive patients, the risk of progression from gingivitis to periodontitis is known to increase in proportion with immune function deterioration and decreased number of CD4 counts [8, 11].

Although there have been a few previous reports of gingival candidiasis developing in the solid cancer patient [7], our case is of interest in that gingivitis progressed into periodontitis resulting permanent alveolar bone damage. According to the literature, the rate as well as severity of periodontal destruction is increased in proportion with the reduction in number of functional polymorphonuclear leukocytes (PMNs) [12]. There are emerging data indicating that, to evade and compromise host immune surveillance, cancer cells utilize various strategies, one of which is mediated by several tumor derived soluble factors such as interleukin-10 (IL-10), transforming growth factor *β* (TGF *β*), and vascular endothelial growth factor (VEGF) [13]. Moreover, while the patients suffer from poor oral intake due to chemotherapy related complications such as nausea, vomiting, or oral mucositis, the nutritional impairments further exacerbate their immune function. Given both the occurrence of gingival candidiasis and progression of gingivitis to periodontitis resulting in bony destruction, we can postulate that his immune function was deteriorated.

One of the important issues to be addressed is how to detect and manage the oral mucositis and gingival candidiasis. According to the guidelines multinational association of supportive care in cancer/International Society of Oral Oncology (MASCC/ISOO) published and updated, careful oral investigation at each patient visit and frequent use of gargle are generally considered as the standard treatment [14]. Even though the patient was treated with the oral care as protocol, there was no improvement in the oral lesion. Two weeks later, gingival candidiasis was confirmed by incision biopsy. Although antifungal therapy was initiated right after diagnosis, bony destruction progressed, which makes the patient suffer excessive pain for the rest of life. In addition, economic and time costfor subsequent visit to dentist for tooth extractions and prosthodontic rehabilitation was also a big burden for the patient. Earlier suspicion and invasive diagnostic approach would have brought better outcomes, although there is no clear evidence at present that initiation of antifungal therapy in early phase of gingival candidiasis can prevent its progression into periodontitis and its long term sequela.

Regarding this patient presented here, several points should be further addressed. First, it would be better to provide information which can show the baseline dental status to compare. Unfortunately, prechemotherapy dental evaluation was not performed for this patient. Hence, it was not possible to precisely assess the influence of chemotherapy on his dental health. Second, although candida species were identified in the incisional biopsy and certainly may have contributed to the pathogenesis of periodontitis, other periodontopathogenic microorganisms may also have played a role. Since no microbiological assessment (cultures/PCR) has been performed, we could not rule out the possibility of potential interplay of other microorganisms such as bacteria or virus and could not find out which Candida subspecies were involved, either. Given the similarity between this case and acute necrotizing gingivitis/periodontitis, it should be considered as another potential differential diagnosis. Also, as recent evidence reports the association between bevacizumab and periodontitis [15], bevacizumab may contribute to this complication.

To the best of our knowledge, this is the first case of gingival candidiasis occurring in the solid tumor patients reported in Korean PubMed. By this case report, we intend to emphasize the immune compromising effect of cancer, especially when they are treated with the cytotoxic chemotherapy agents. Moreover, given this immunocompromising effect of the conventional cytotoxic chemotherapy, when apparently common side effects were not treated effectively with conservative oral care using gargle, the rarer complications such as uncommon infection should be suspected earlier, and more aggressive diagnostic tools including surgical biopsy should be considered in the early phase of the disease to stop its progression.
